# Supplementary material for: Selective cleavage of ncRNA and antiviral activity by RNase2/EDN in THP1-induced macrophages
Source: Cell Mol Life Sci. 2022 Mar 26;79(4):209. doi: 10.1007/s00018-022-04229-x (PMC8960563; doi:10.1007/s00018-022-04229-x)
Supplement: Supplementary file 1 — Supplementary file1 (DOCX 5552 KB) [file 18_2022_4229_MOESM1_ESM.docx]

# **SUPPLEMENTARY MATERIAL- Figures and Tables**

**Selective cleavage of ncRNA and antiviral activity by RNase2/EDN in a THP1-induced macrophages**

**Lu Lu^1,2#*^, Jiarui Li^1*^, Ranlei Wei^3^, Irene Guidi^1^, Luca Cozzuto^4^, Julia Ponomarenko^4^, Guillem Prats-Ejarque^1^ and Ester Boix^1#^**

^1^Department of Biochemistry and Molecular Biology, Faculty of Biosciences, Universitat Autonoma de Barcelona, Cerdanyola del Vallès, Spain

^2^College of Animal Science and Technology, Sichuan Agricultural University, Chengdu, Sichuan, China

^3^National Frontier Center of Disease Molecular Network, West China Hospital, Sichuan University, Chengdu, 610041, China

^4^Bioinformatic Unit. Centre de Regulació Genòmica (CRG), Barcelona, Spain

**^*^**Both authors contributed equally to this work

**^#^** Corresponding authors: Ester Boix, [Ester.Boix@uab.es](mailto:Ester.Boix@uab.es) ; Lu Lu, [lu.lu@sicau.edu.cn](mailto:lu.lu@sicau.edu.cn)

**Supplementary figures**


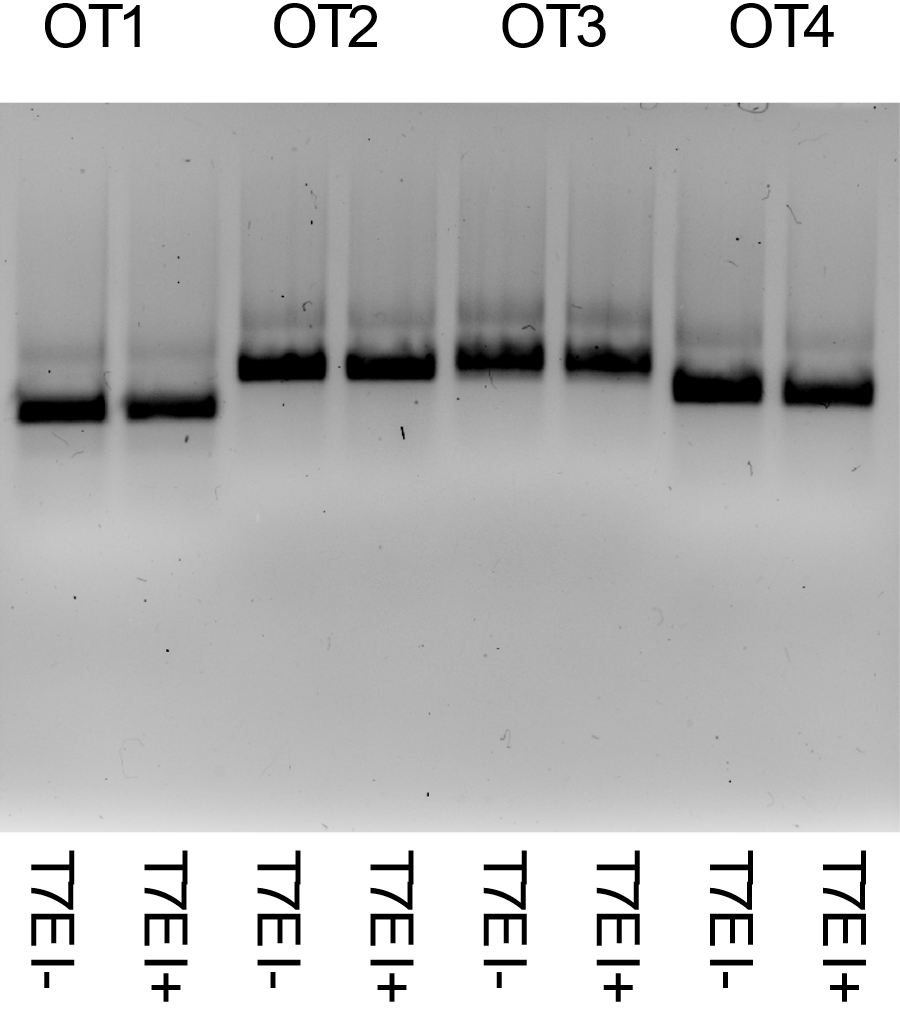


**S1 Fig. Analysis of the potential off-target effects of sgRNA1.** The sgRNA1 mediated off-target sites OT1-4 were predicted and analysed by T7EI assays.

**

S2 Fig. Titration of RSV in THP1-derived macrophages from 0 to 72h post of inoculation.** The intracellular **(A)** and extracellular **(B)** RSV was quantified by probe qPCR and calculated as the median tissue culture infectious dose (TCID_50_); the stars refer to the significance between KO+ and WT+, “+” and “-” indicate with or without RSV infection, respectively. Significance is indicated (* *p* < 0.05 and ** *p* < 0.01).

**
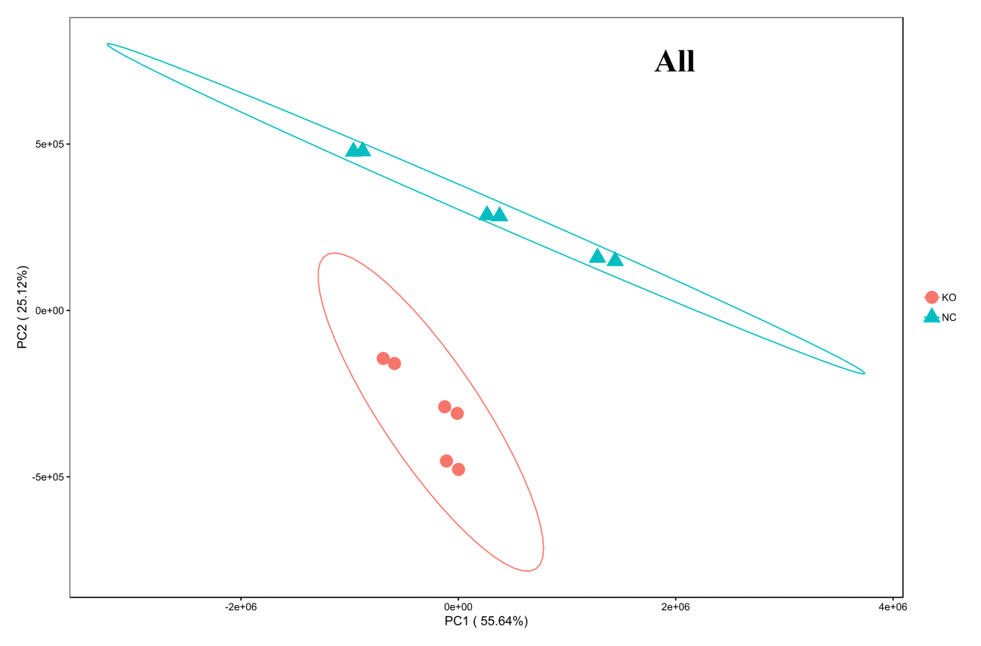
**

**S3 Fig. Principal analysis component (PCA) for THP1 WT and RNase2-KO for all ncRNA analysis by Cp-RNAseq.**


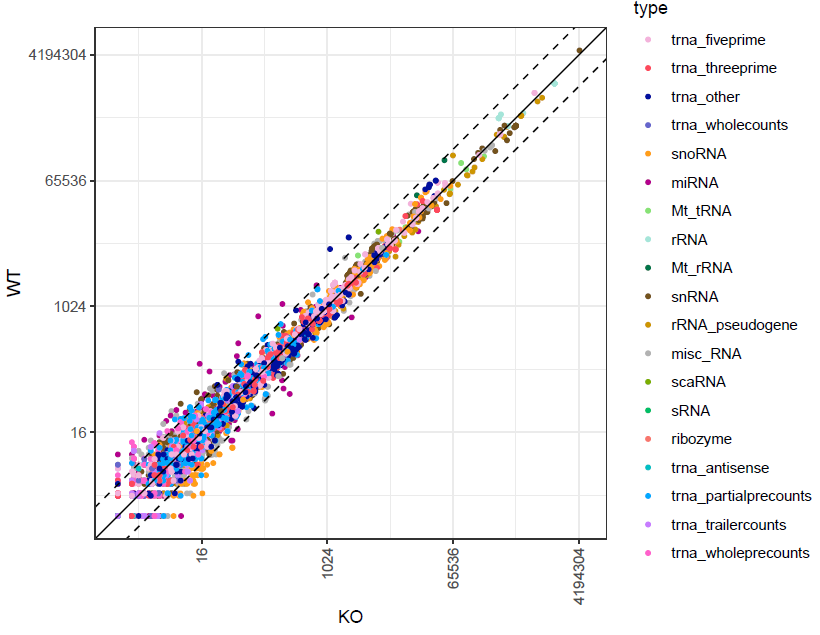
**S4 Fig. Small RNA type scatter plot for WO vs RNase2-KO samples**. For a full list of small RNAs identified by cP-RNAseq and statistics see Additional file 1.


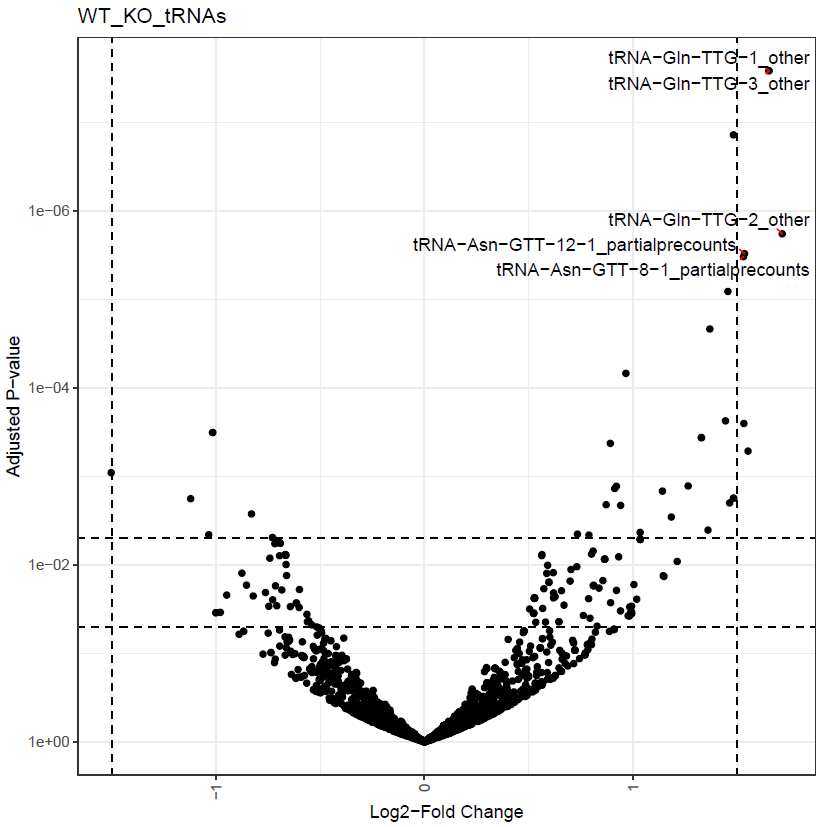


**S5 Fig. Volcano Plot for tRNAs and tRNA-derived fragments in THP1-WT vs KO cells.** The significantly down-regulated and up-regulated tRNA fragments identified by cP-RNAseq are shown (cut-off values of Log2FoldChange and padj are indicated). The top significant tRNA fragments are labelled with their name. See Additional file 2 for a complete list of sequences and statistics.


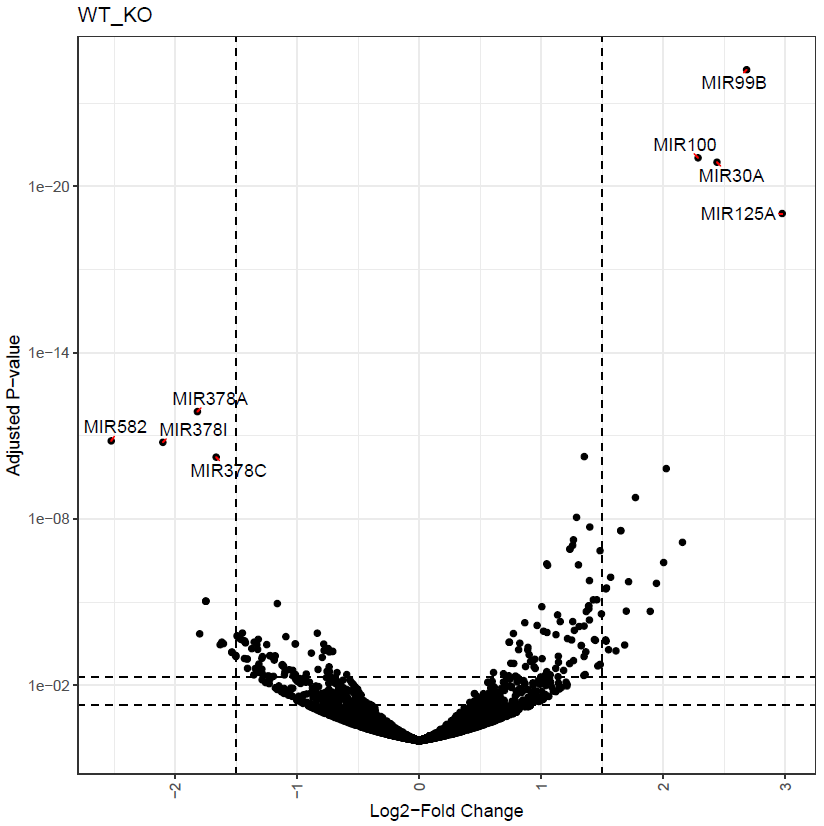


**S6 Fig**. **Volcano plot for miRNAs abundance in THP1-WT vs KO cells identified by cP-RNAseq methodology.** See Additional file 3 for a complete list of sequences and statistics.


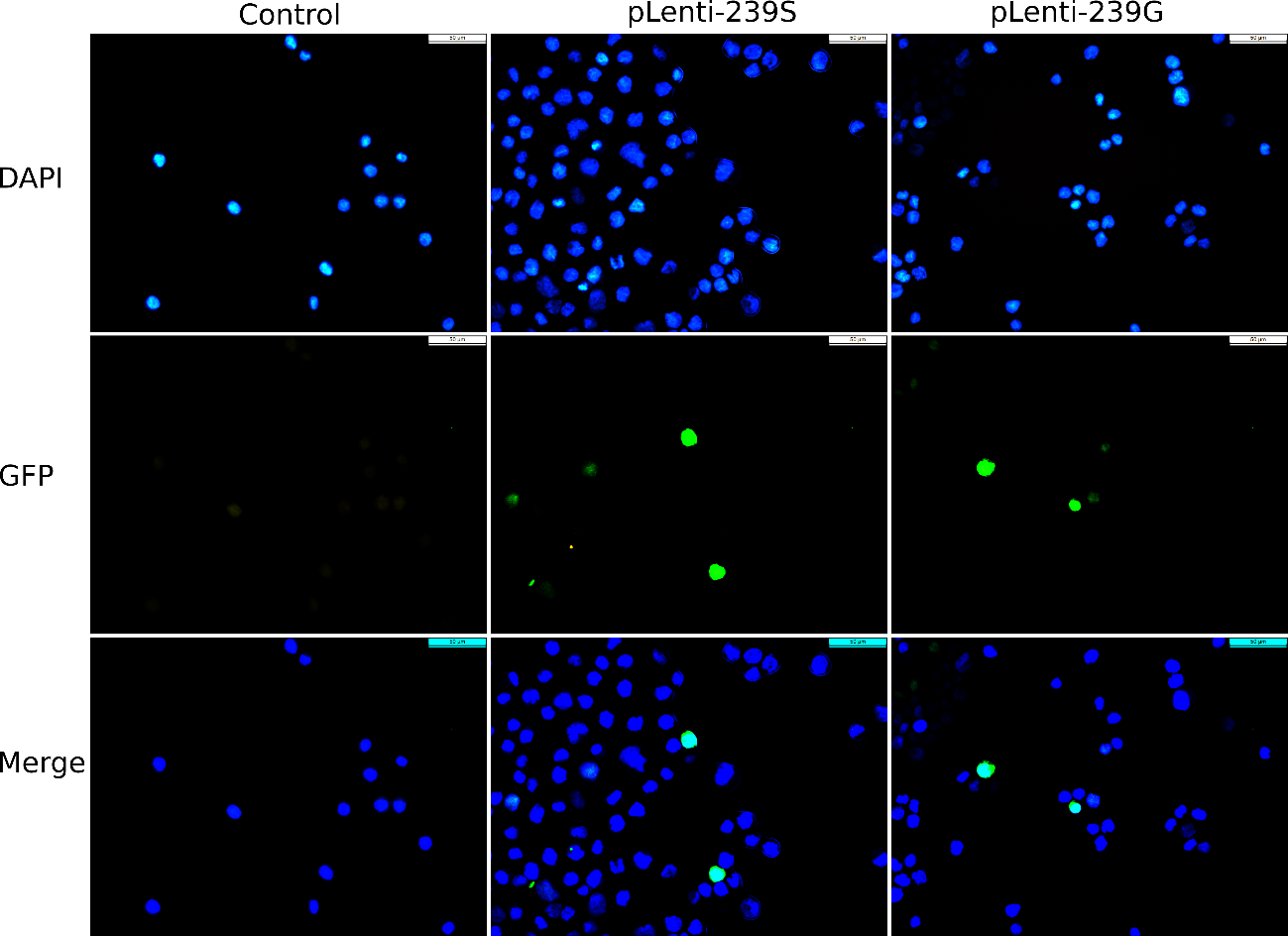


**S7 Fig. Representative image of THP1 cells transduced by CRISPR lentiviral.** 1x10^6^ of THP1 cells were resuspended in 2mL of RPMI+10%FBS and incubated in 37 ℃ at 5% CO_2_ for 2h before the transduction. 20 µl of the concentrated virus and 8 μg/mL polybrene were added into THP1 culture in 6-well plate. After overnight’s culture, the medium was changed with fresh medium and the cells were continually grown. After 72h post of the infection, the cell samples were fixed by 4% paraformaldehyde and dyed with DAPI and the fluorescence images were captured using Olympus BX50 fluorescence microscopy. The GFP positive cells were calculated to evaluate the transduction efficiency of lentiviral with THP1 cells from 15 randomly chosen images.


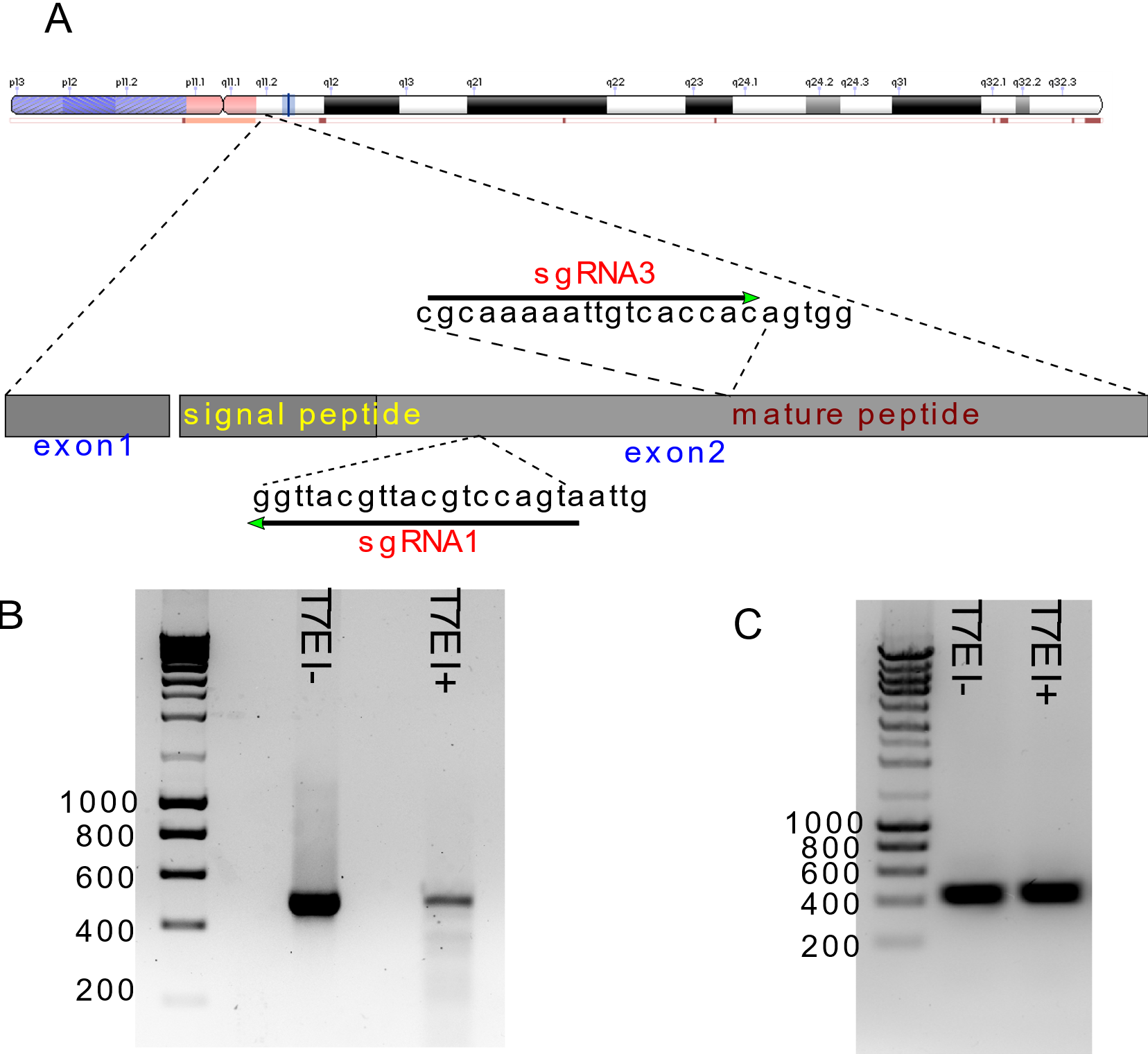


**S8 Fig. Evaluation of the knockout efficiency of sgRNA1-6 targeting the AAVS1 locus in HeLa cells. A)** Schematic of sgRNA1&3 designed to target the RNase2 locus; **(B)** and **(C)** Detection of the knockout efficiency of sgRNA1 and sgRNA3 using a T7E I assay, respectively. Amplicons of sgRNA1&3 that were treated with T7E I (T7E I+) or without T7E I (T7E I+) were separated by 2% agarose gel electrophoresis. DSB sites were recognized and digested by T7E I. Undigested and digested bands were consistent with the predicted sizes from the RNase2 locus.


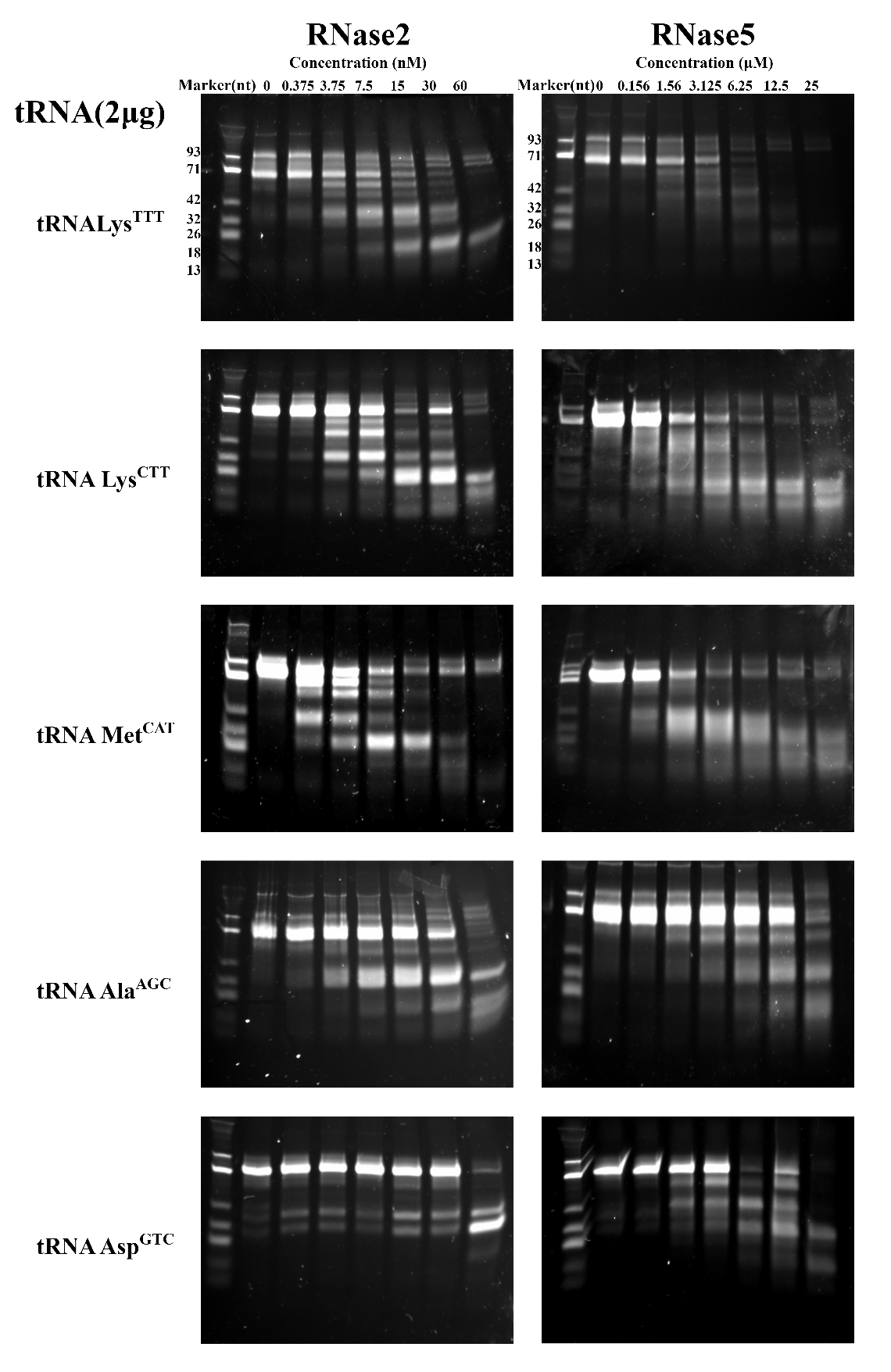


**S9 Fig. *In vitro* analysis of degradation products of representative tRNAs by RNase2 and RNase5.** The tRNA degradation products released by RNase2/RNase5 cleavage were separated by 12% urea-PAGE electrophoresis. The gel was visualized under ultraviolet light after staining with Sybr Gold.

**Supplementary tables**

**S1 Table. Oligos of the sgRNAs and sequencing primers**

| **Target name** | **Sequence** |  |
| --- | --- | --- |
| SgRNA1 | GTTAATGACCTGCATTGCATTGG |  |
| sgRNA3 | CGCAAAAATTGTCACCACAGTGG |  |
| **Off-target name** |  | Chromosome location |
| OT-1 | GTTAATGACCTGCATTGCATTGG | chr10:116445782-116445804:+ |
| OT-2 | CTTAATGATATGCATTGCAT AGG | chr4:95875569-95875591:- |
| OT-3 | GATAATGAGATACATTGCAT TGG | chr2:211644633-211644655:+ |
| OT-4 | TTTTATGACCTGTATAGCAT AGG | chr6:22605178-22605200:+ |
| **PCR primers** |  |  |
| sgRNA-x-Fw | TCTTCTGTTGGGGCTTCTGGCTG | 482 |
| sgRNA-x-Rv | GATGAGTGATGATGAGGAGTGCT |  |
| sgRNA3-Fw | TCTTCTGTTGGGGCTTCTGGCTG | 482 |
| sgRNA3-Rv | GATGAGTGATGATGAGGAGTGCT |  |
| OT1-Fw | TCAGGGGCTAAAATAGGGTGC | 107 |
| OT1-Rv | TATTGGATTGCCTGCTCCCC |  |
| OT2-Fw | ACAAACTCTGCAGCTTTGGC | 123 |
| OT2-Rv | TCTATGTAAACAGAGTCTTTGGCA |  |
| OT3-Fw | AGGGGCTTGACTCAGAGAGT | 129 |
| OT3-Rv | GGCATGCATCCATTGAGCTG |  |
| OT4-Fw | TCCATGTAATCTGCCAGCCA | 115 |
| OT4-Rv | AATTGGGTGGCTGAGGTGAC |  |
| **Sequencing primers** |  |  |
| U6-seq | GAGGGCCTATTCCCATGATT |  |
| Fw-seq | GCTTCTTCTGTTGGGGCTTC |  |
| Rv-seq | TCAACGACGAGACCCTCCAC |  |

**S2 Table. List of parental tRNAs with significant differential coverage between WT and RNase2-KO macrophage cells.** Predicted cleavage sites of the most significantly abundant tRNA-derived fragments identified by cP-RNAseq (see Additional file 2 for a full list) is indicated. Only sequences with a log_2_fold >0.5 and *p* adjusted < 0.05 are included.

| **Classification** | **log_2_**  **(WT/KO)** | ***p***  **(WT/KO)** | **Main predicted cleavage site** | **Differential coverage between WT (black) and KO (grey)** |
| --- | --- | --- | --- | --- |
| tRNA-Ala-AGC-4 | 0.53 | 0.0239 | GU AG | 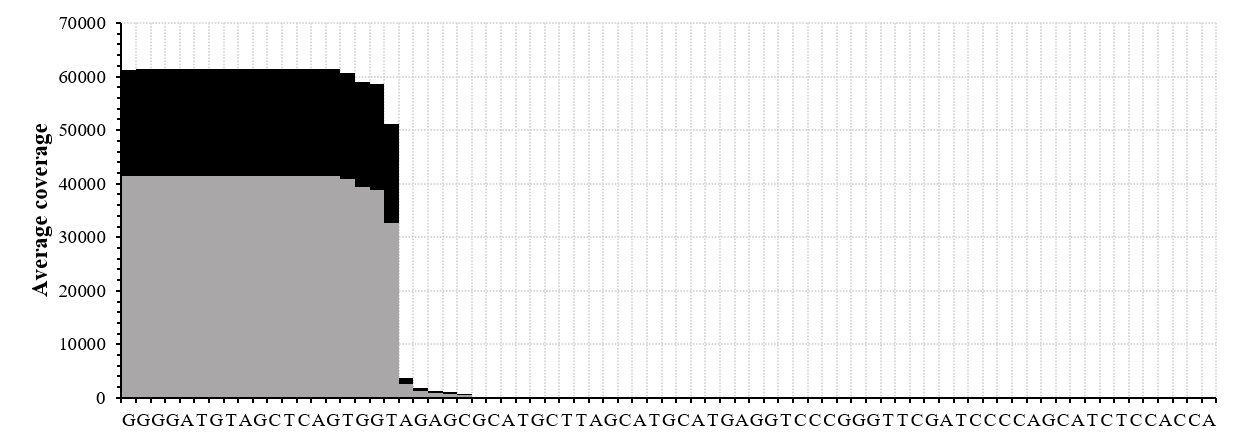 |
| tRNA-Ala-CGC-1 | 0.53 | 0.0239 | GU AG | 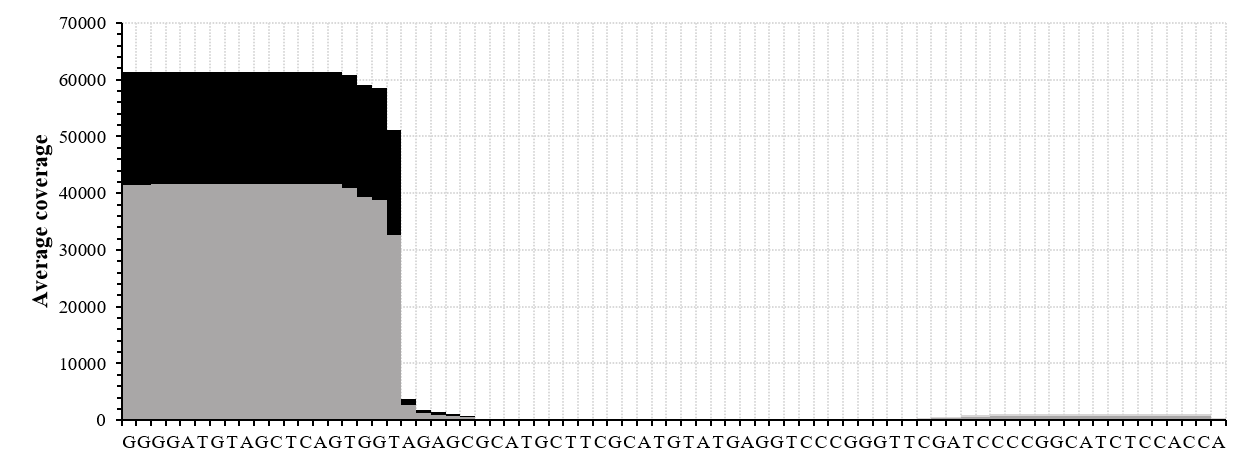 |
| tRNA-Ala-CGC-2 | 0.53 | 0.0239 | GU AG | 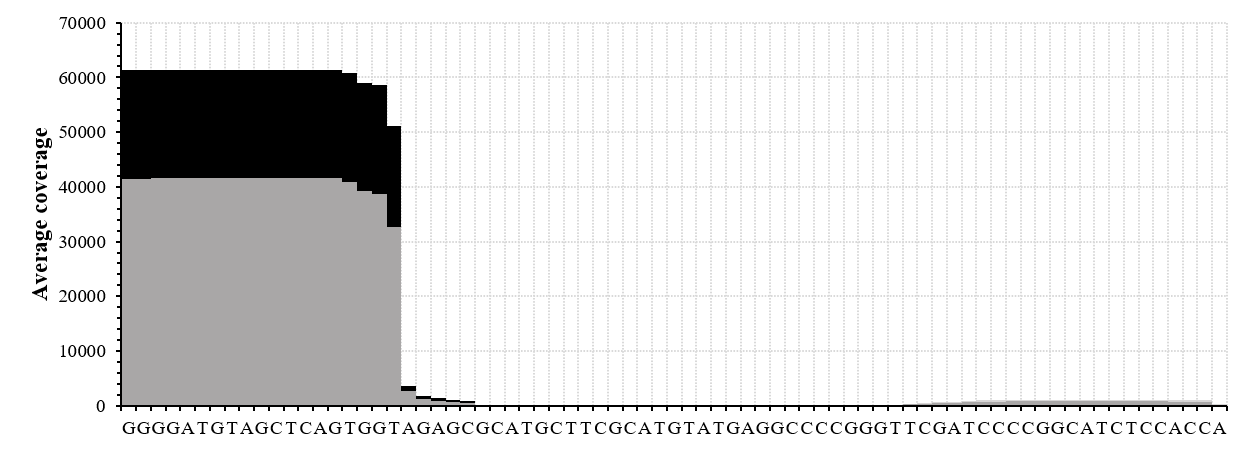 |
| tRNA-Ala-CGC-3 | 0.53 | 0.0240 | GU AG | 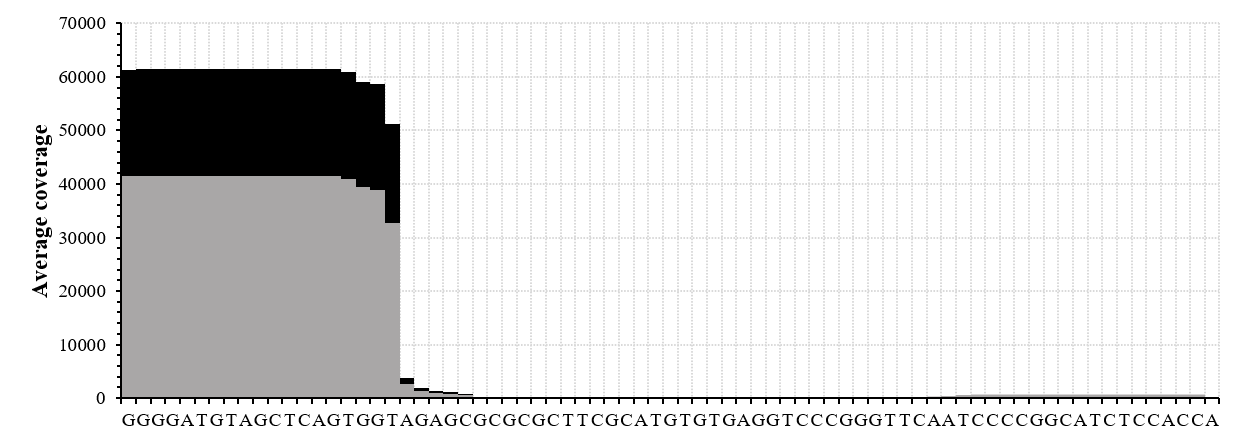 |
| tRNA-Ala-TGC-2 | 0.53 | 0.0239 | GU AG | 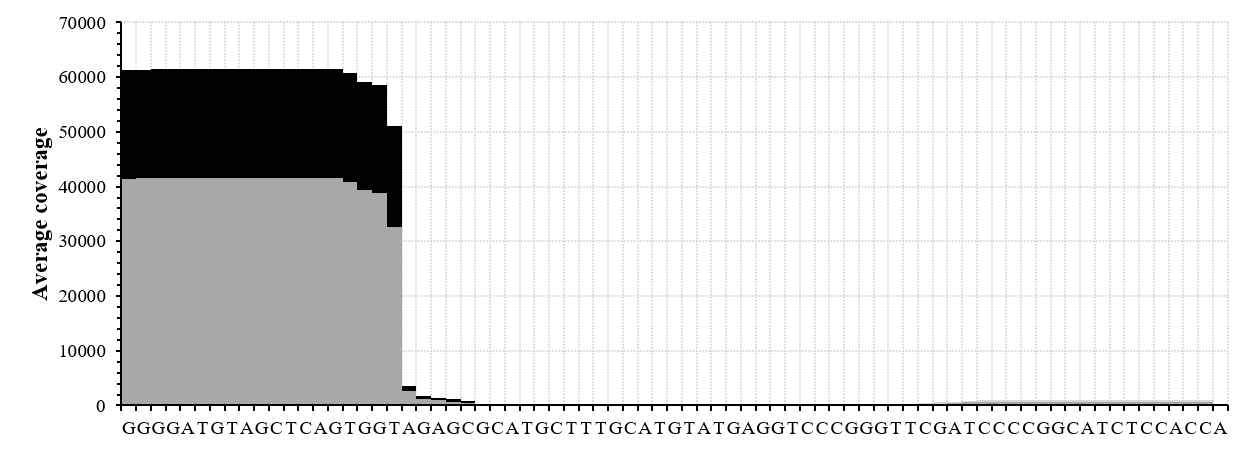 |
| tRNA-Ala-TGC-3 | 0.53 | 0.0239 | GU AG | 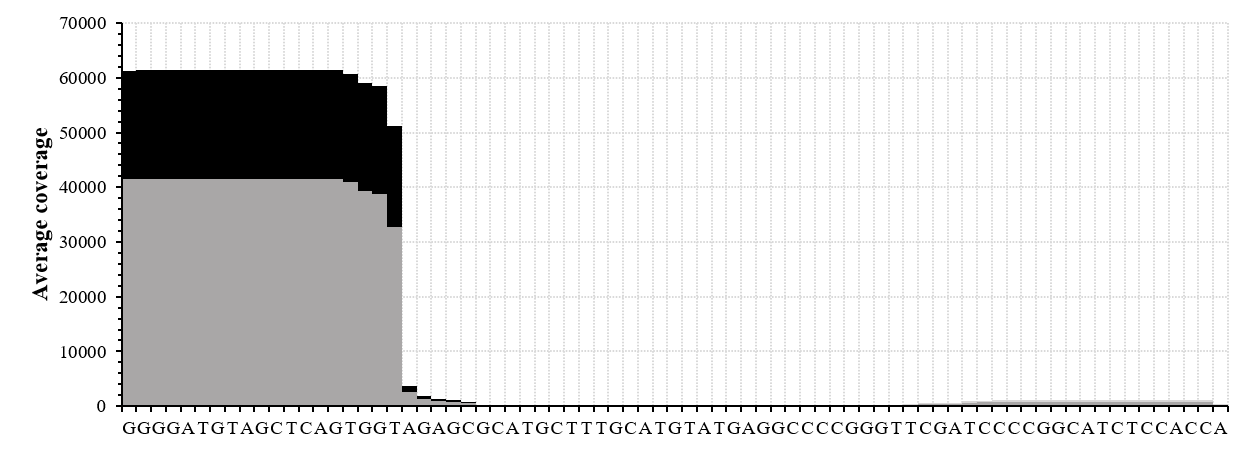 |
| tRNA-Ala-TGC-4 | 0.53 | 0.0235 | GU AG | 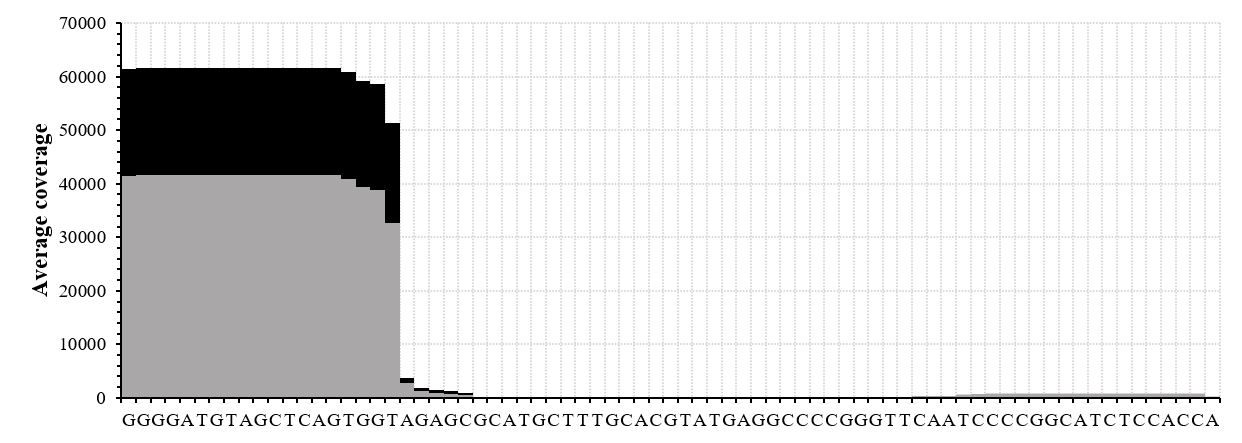 |
| tRNA-Asn-GTT-8 | 0.60 | 0.0159 | GA UC | 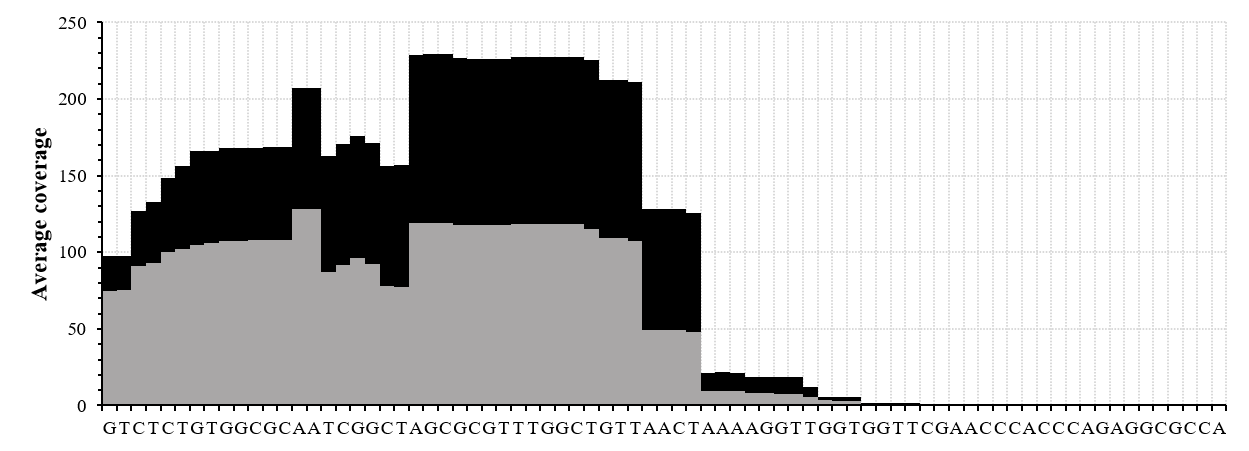 |
|  | 0.73 | 0.0105 | CU AA |  |
| tRNA-Asn-GTT-10 | 0.62 | 0.0123 | GA UC | 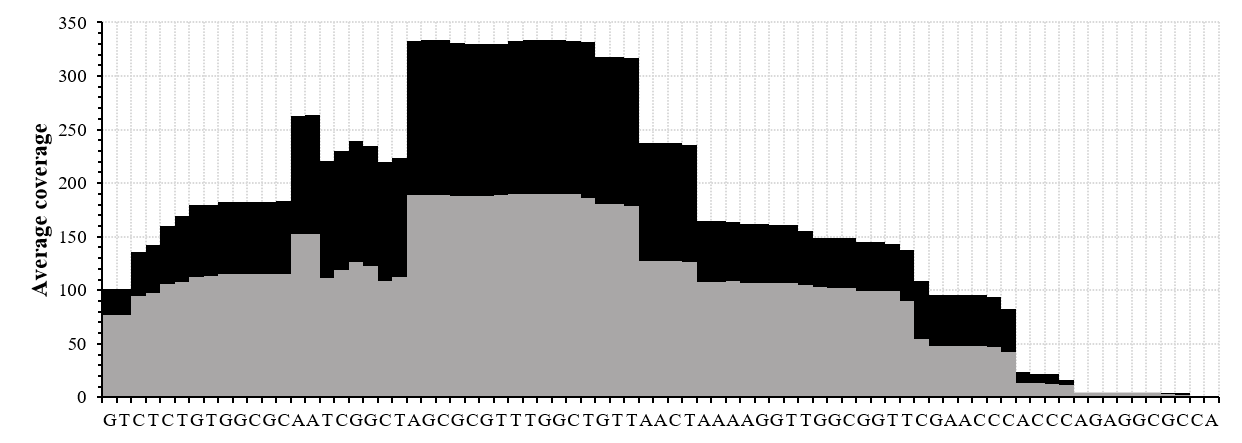 |
|  | 0.62 | 0.0237 | CU AA |  |
| tRNA-Asn-GTT-12 | 0.60 | 0.0157 | GA UC | 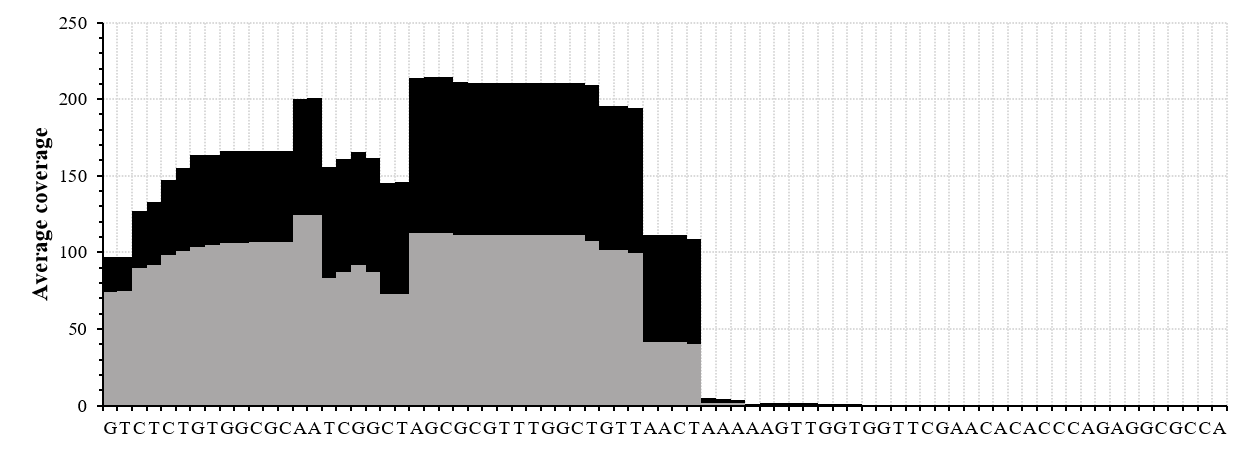 |
|  | 0.70 | 0.0153 | CU AA |  |
| tRNA-Asp-GTC-4 | 0.67 | 0.0286 | CA CU | 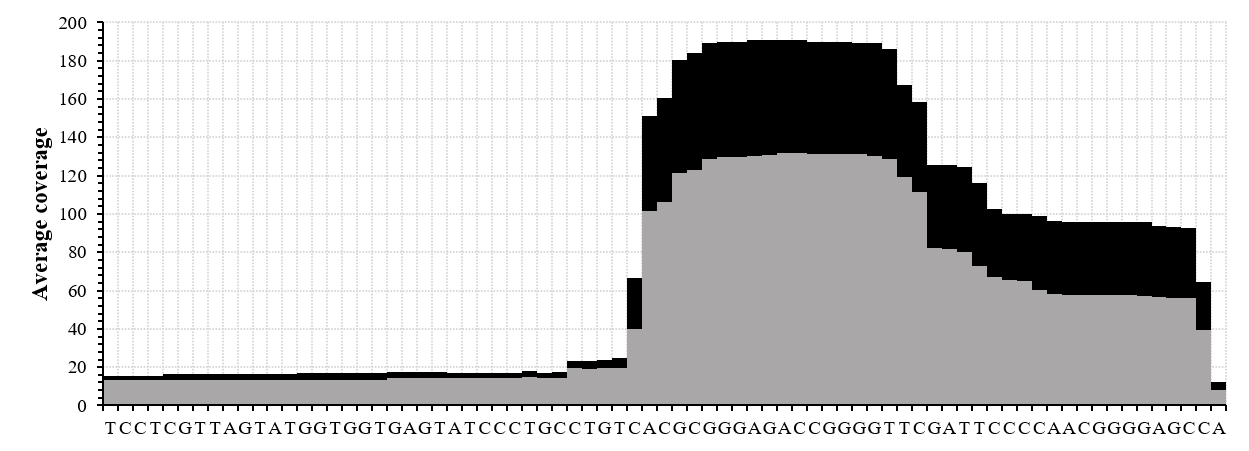 |
| tRNA-Asp-GTC-5 | 0.92 | 0.0195 | GU CA | 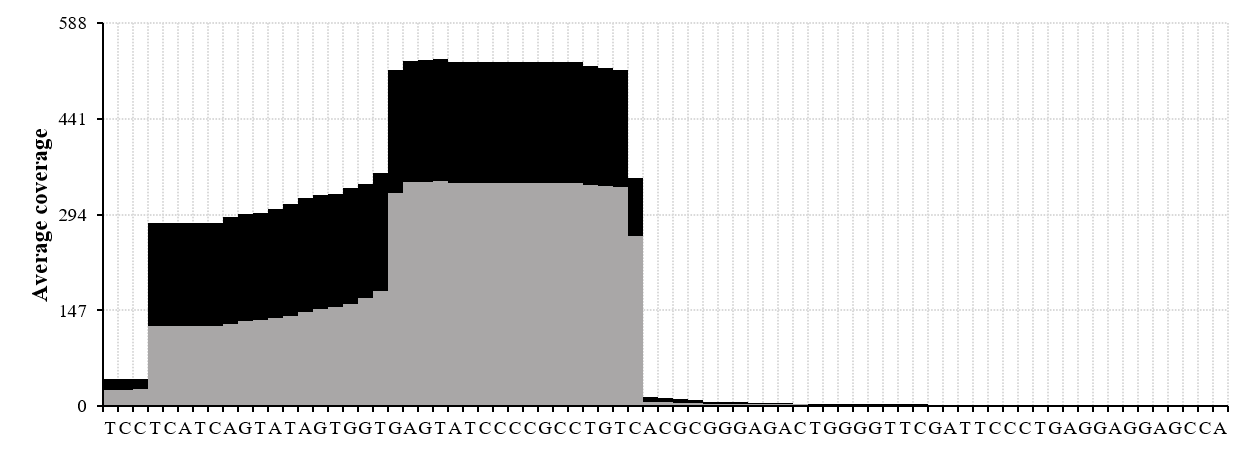 |
| tRNA-Cys-GCA-1 | 0.59 | 0.0101 | GC AG | 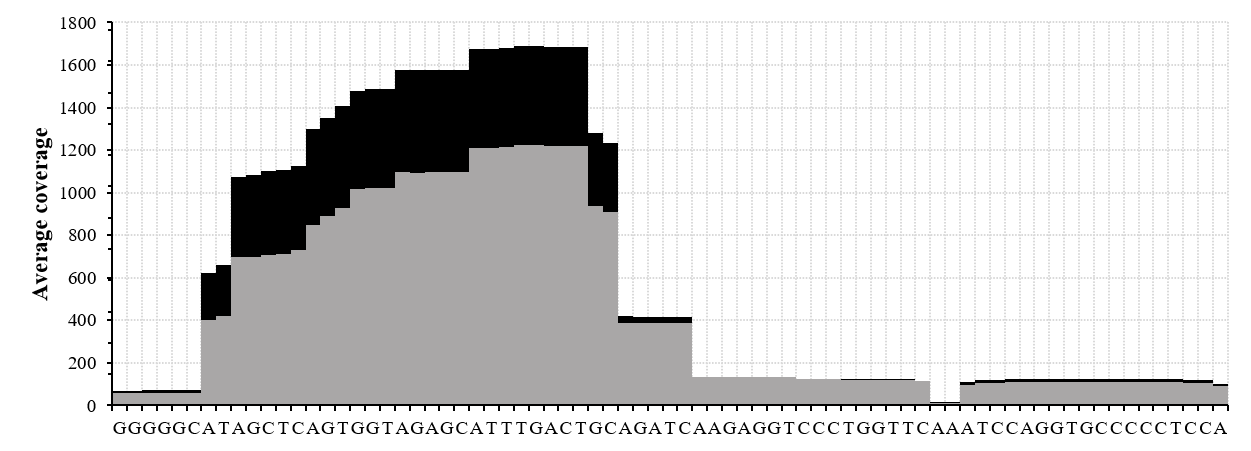 |
| tRNA-Cys-GCA-2 | 0.56 | 0.0079 | GC AG | 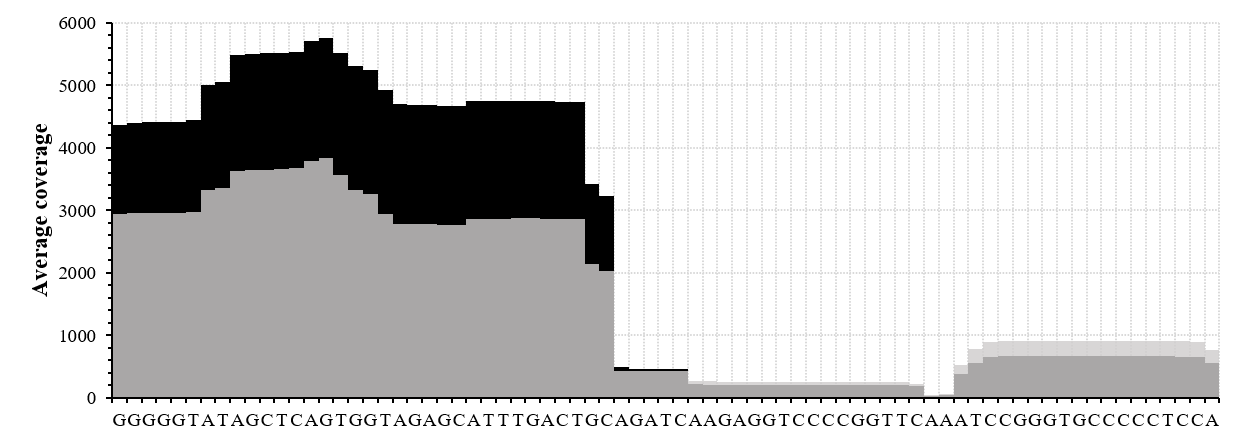 |
| tRNA-Cys-GCA-4 | 0.56 | 0.0077 | GC AG | 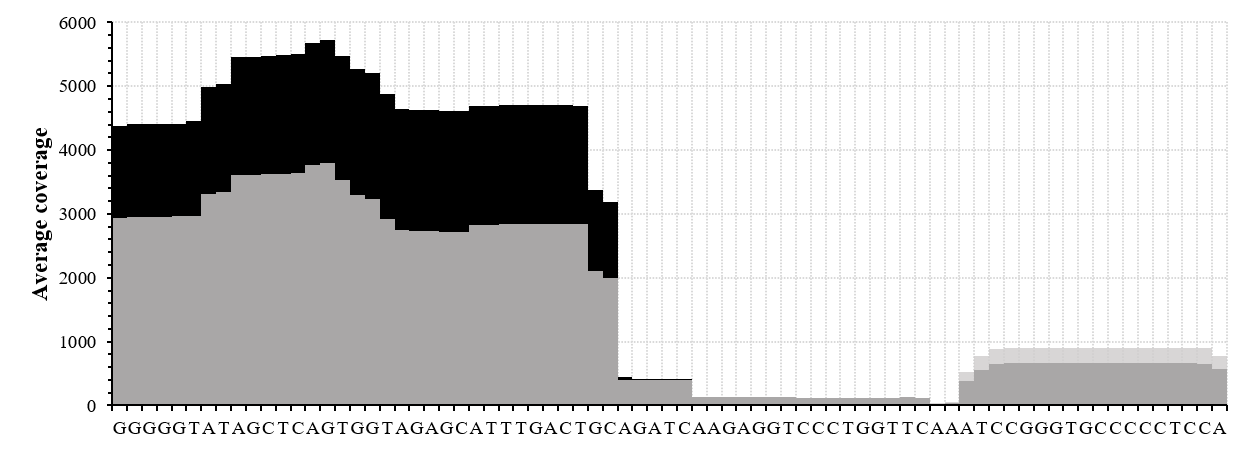 |
| tRNA-Gln-CTG-3 | 1.26 | 0.0013 | CC AG | 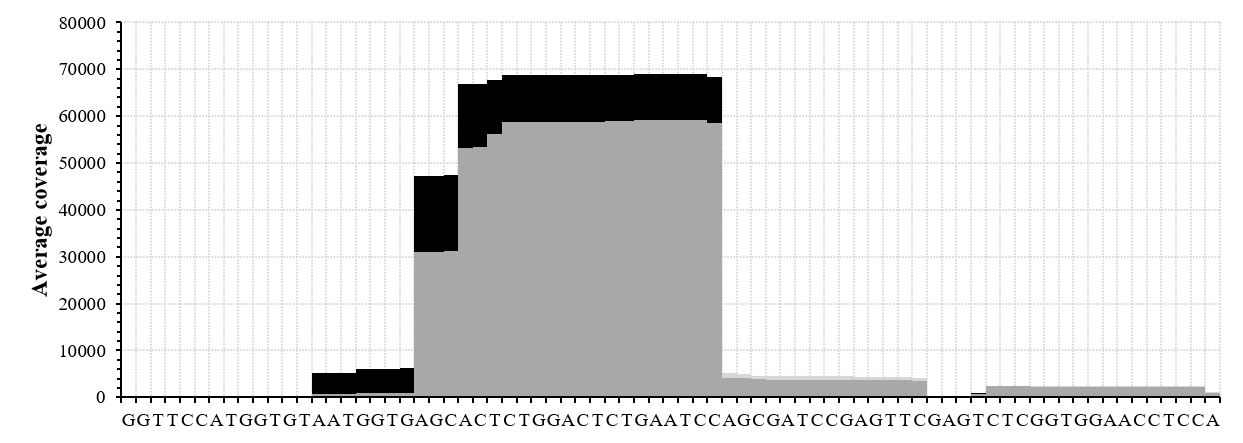 |
| tRNA-Gln-CTG-8 | 1.03 | 0.0043 | AC AG | 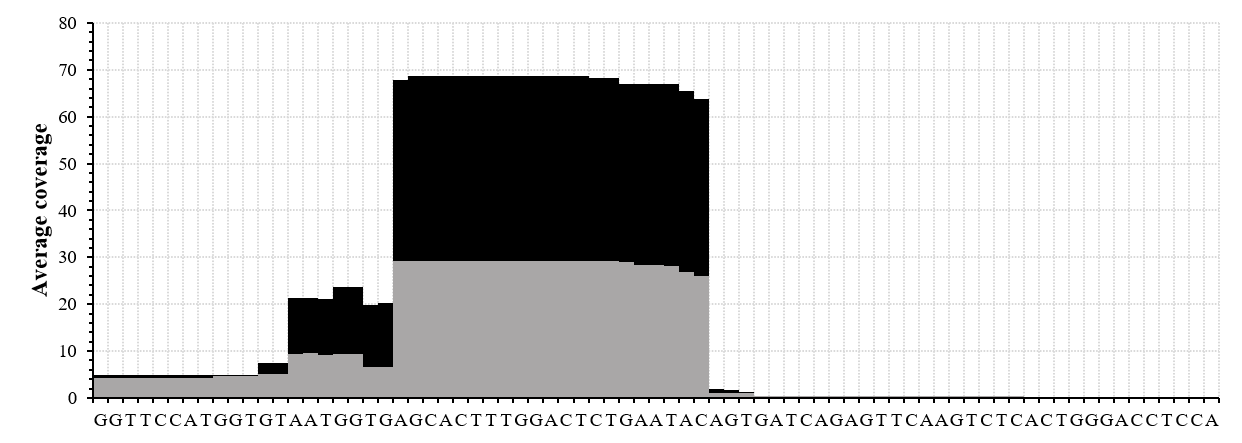 |
| tRNA-Gln-TTG-1 | 1.65 | 2.6300E-08 | CC AG | 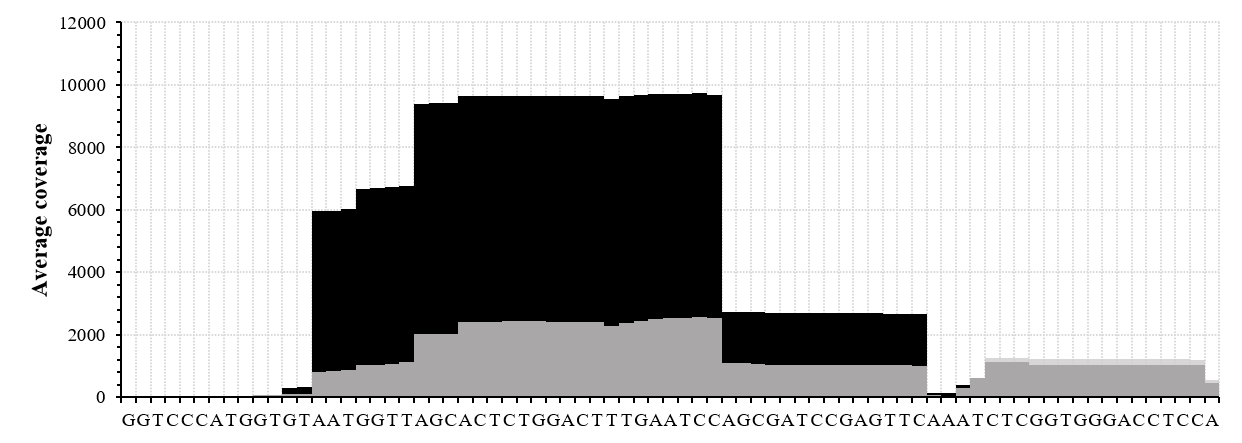 |
| tRNA-Gln-TTG-2 | 1.72 | 1.8300E-06 | CC AG | 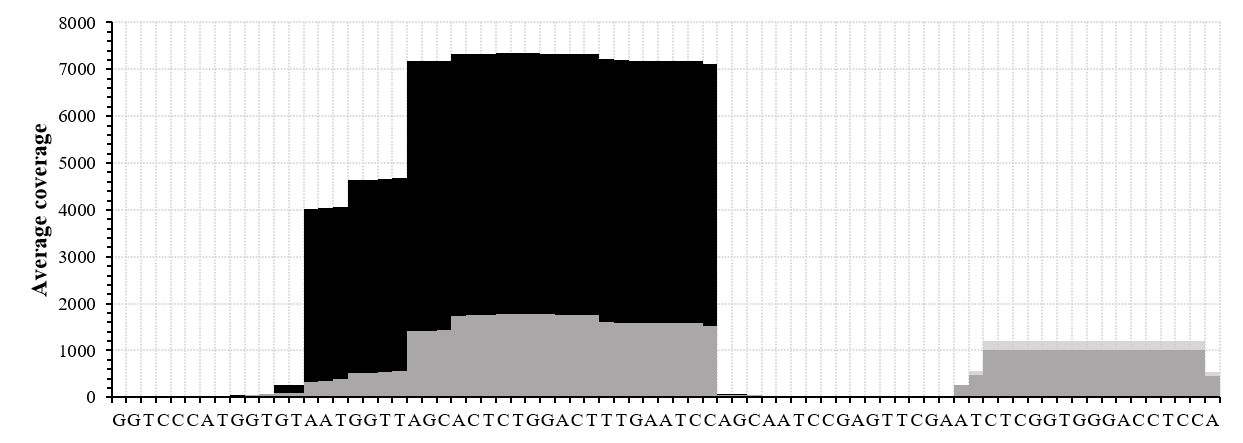 |
| tRNA-Gln-TTG-3 | 1.65 | 2.6300E-08 | CC AG | 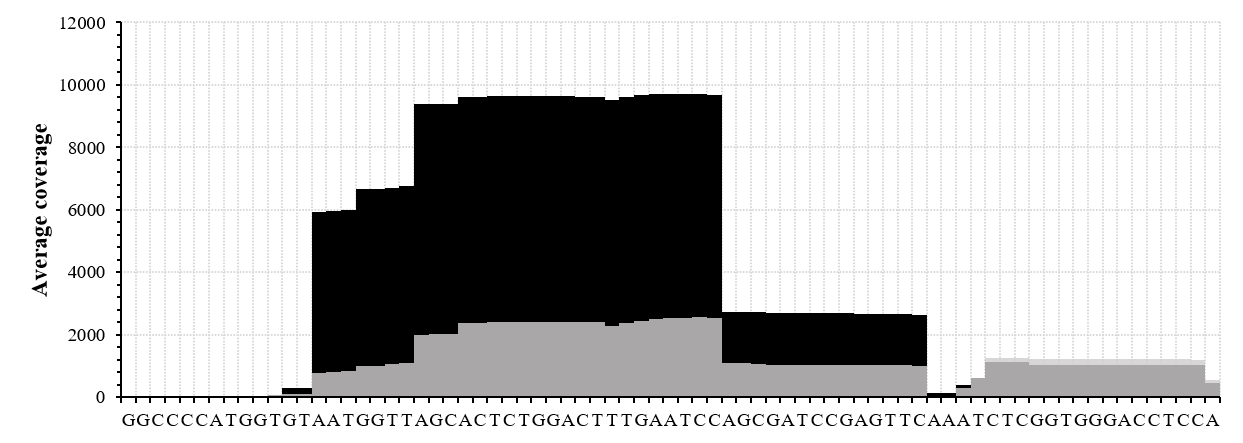 |
| tRNA-Glu-TTC-4 | 0.53 | 0.0447 | CA CU | 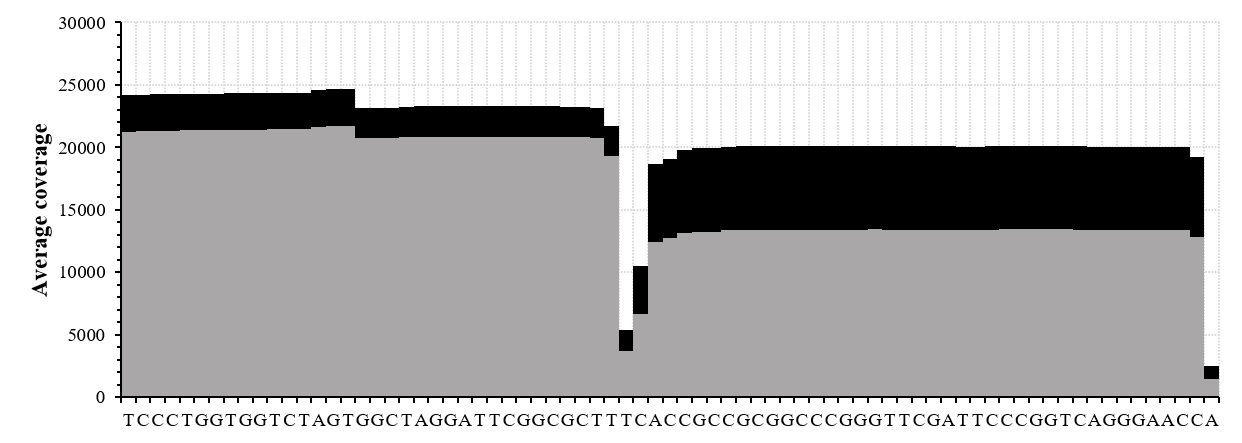 |
| tRNA-Tyr-GTA-11 | 0.63 | 0.0229 | GU AG | 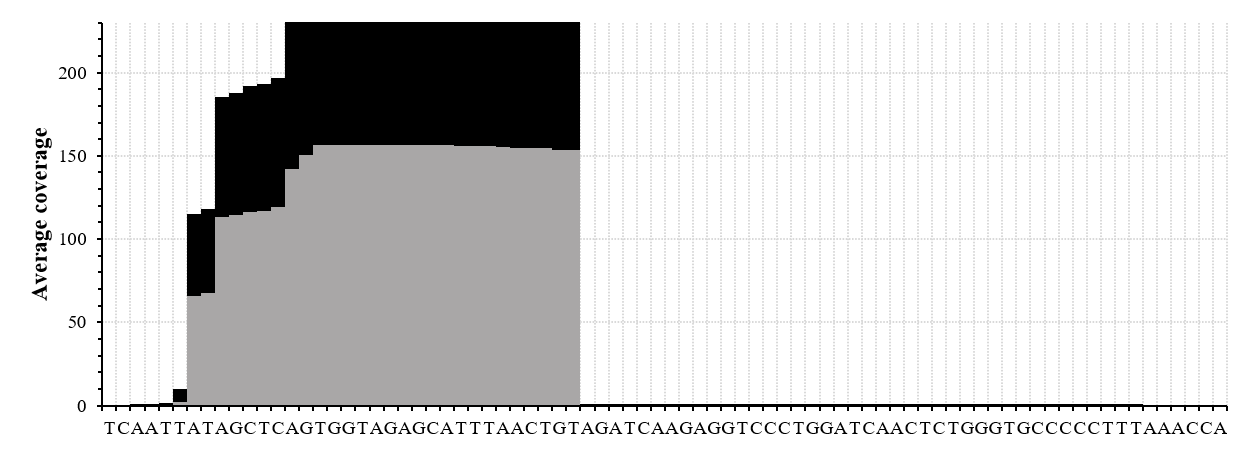 |

**S3 Table. Over-represented miRNA sequences identified in WT vs RNase2-KO THP1 cells. Putative location of cleavage sites is indicated. Information taken from miRBase.**

| **Accession number** | **ID** | **Log_2_ fold change** | ***P* (adjusted)** | **Identified Sequence** | **Main predicted cleavage site** | **Differential coverage between WT (black) and KO (grey)** |
| --- | --- | --- | --- | --- | --- | --- |
| MIMAT0000087 | hsa-miR-30a-5p | 3.04 | 2.6200E-27 | UGUAAACAUCCUCGACUGGAAG | GC UG | 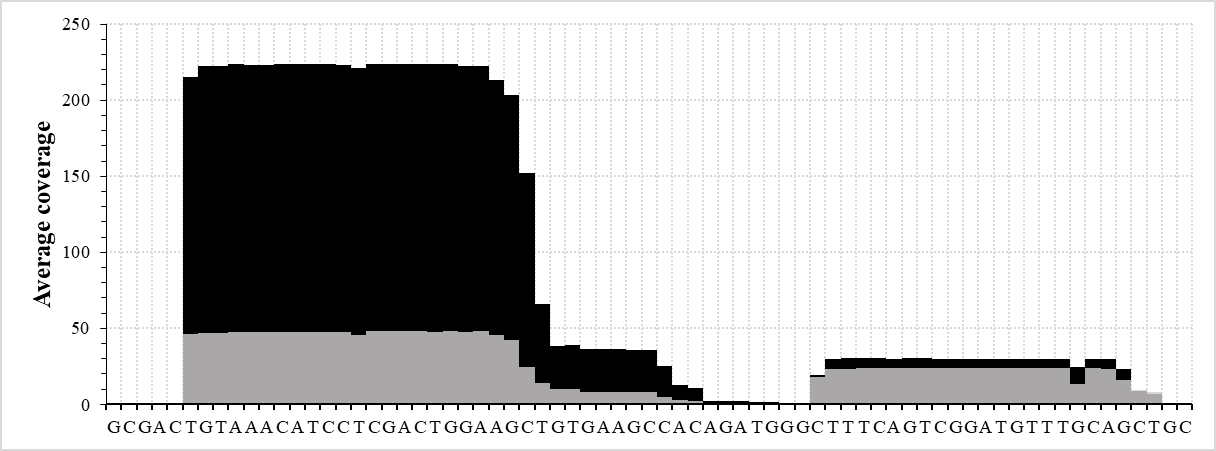 |
| MIMAT0000689 | hsa-miR-99b-5p | 2.85 | 1.5033E-21 | CACCCGUAGAACCGACCUUGCG | GC GG | 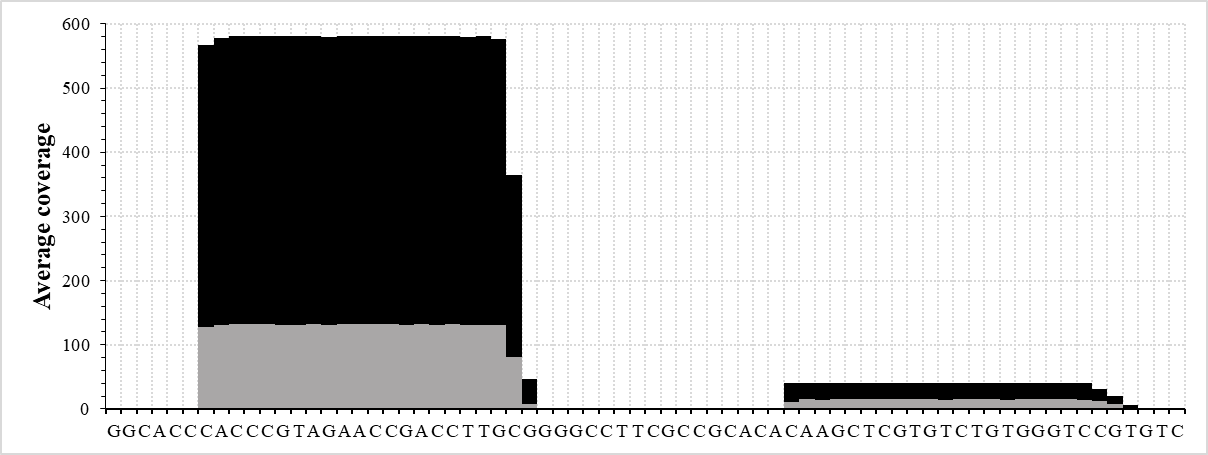 |
| MIMAT0004678 | hsa-miR-99b-3p | 2.21 | 2.7531E-04 | CAAGCUCGUGUCUGUGGGUCCG | CG UG |  |
| MIMAT0000098 | hsa-miR-100-5p | 2.32 | 1.4260E-19 | AACCCGUAGAUCCGAACUUGUG | GU GG | 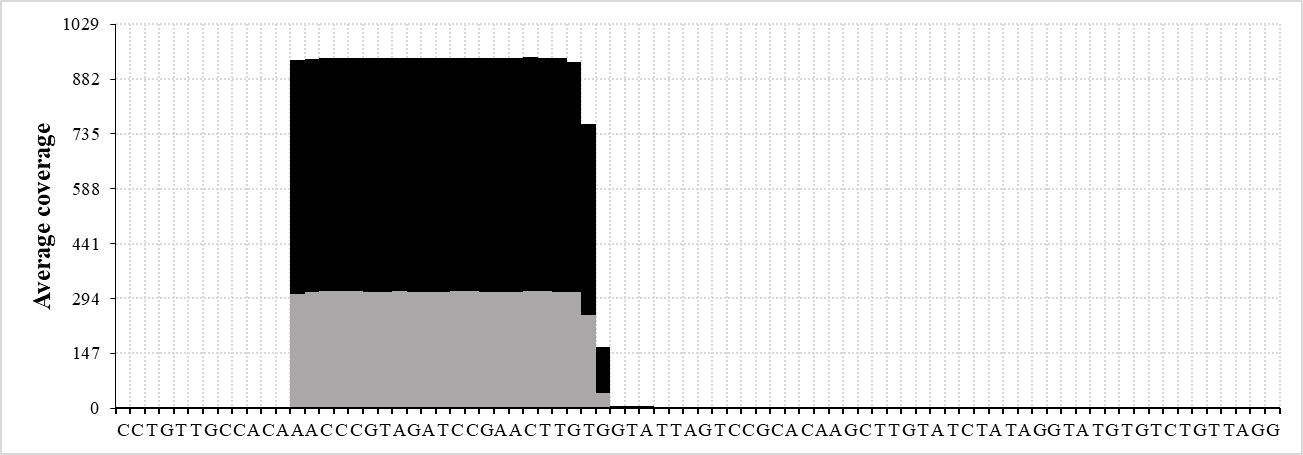 |
| MIMAT0000443 | hsa-miR-125a-5p | 3.33 | 7.8595E-15 | UCCCUGAGACCCUUUAACCUGUGA | UG AG | 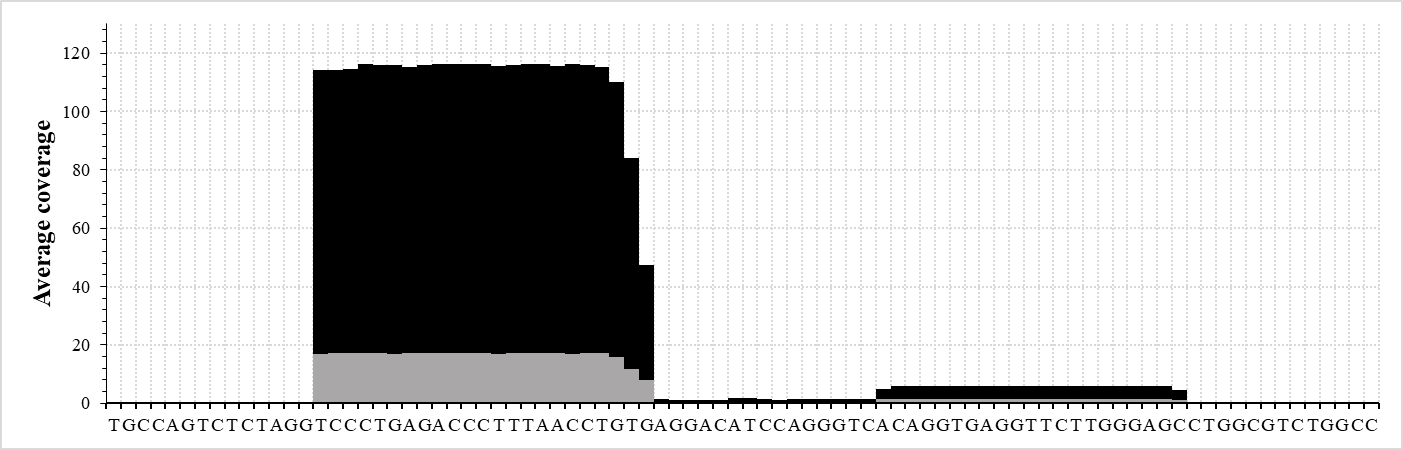 |
| MIMAT0000066 | hsa-let-7e-5p | 2.48 | 4.9694E-10 | UGAGGUAGGAGGUUGUAUAGUU | GU UG | 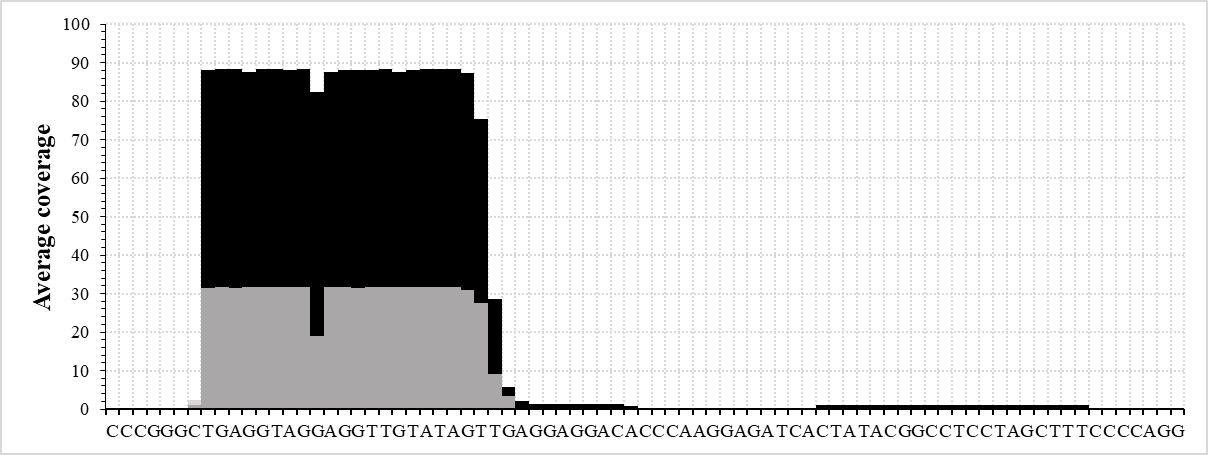 |
| MIMAT0000423 | hsa-miR-125b-5p | 2.40 | 6.0555E-05 | UCCCUGAGACCCUAACUUGUGA | UG AU | 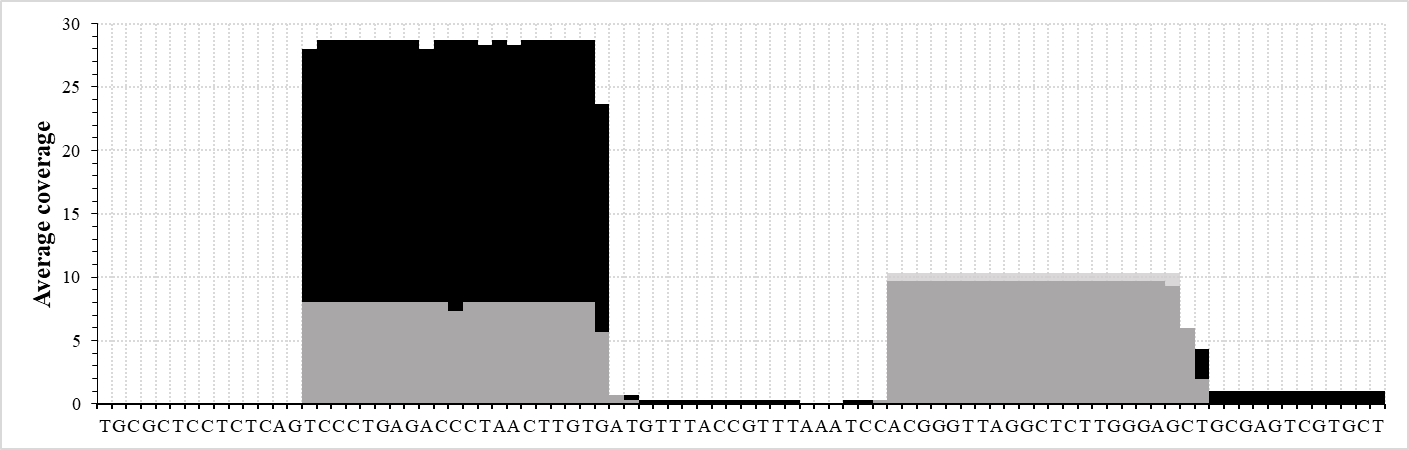 |
|  |  |  |  |  | UG AG | 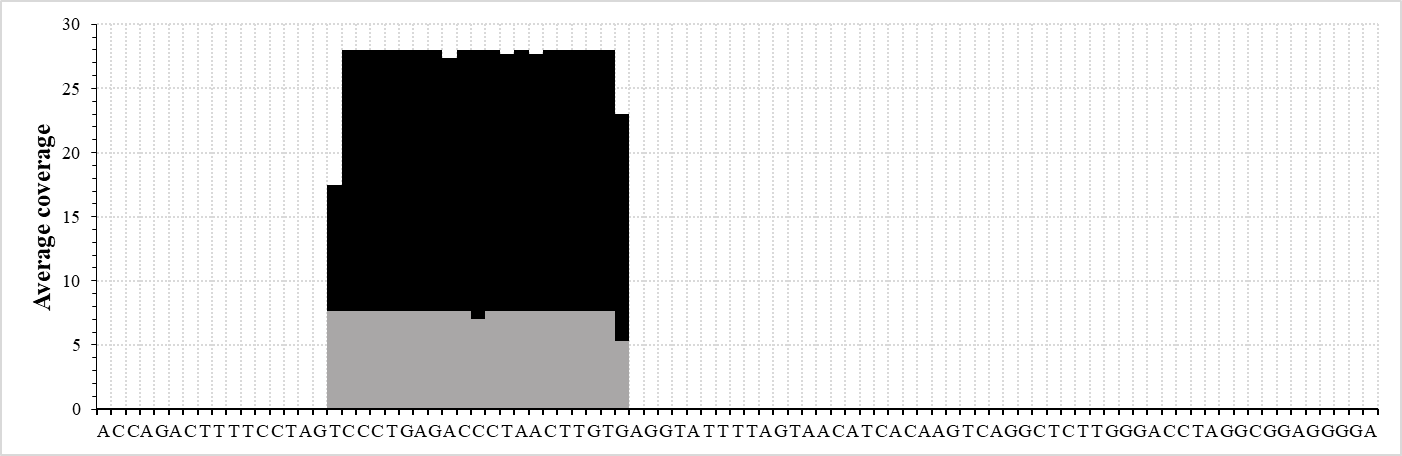 |
| MIMAT0003150 | hsa-miR-455-5p | 2.76 | 3.8997E-03 | UAUGUGCCUUUGGACUACAUCG | GU GG | 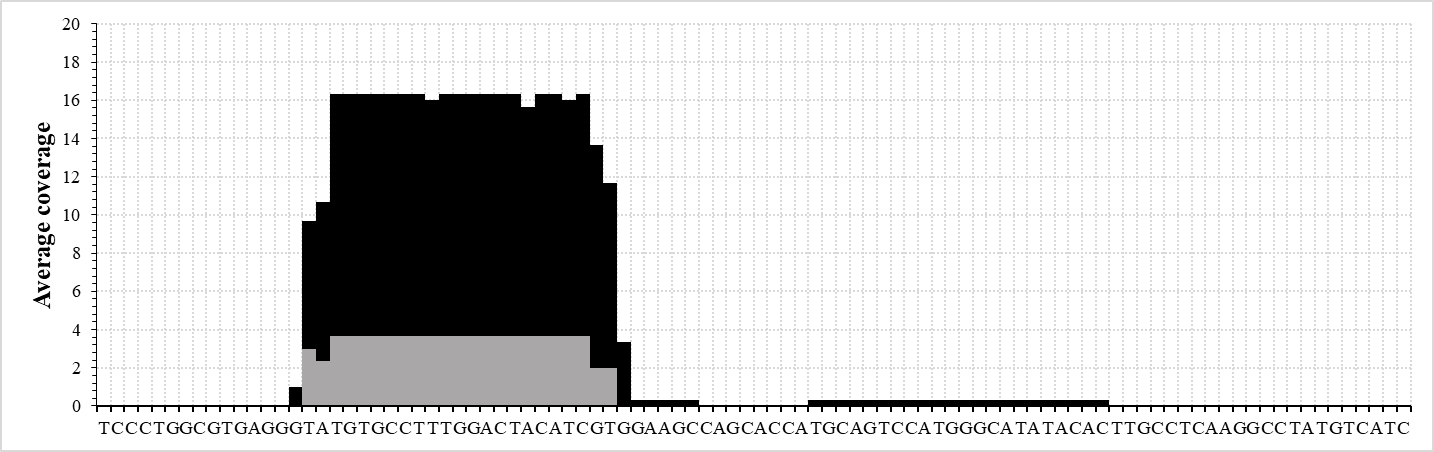 |

**S4 Table. List of tiRNA & tRFs significantly overrepresented in WT vs RNase2-KO THP1 cells. The identified sequences and potential cleavage regions are shown.**

| **Condition** | **Transcript name** | **Parental tRNA** | **Type** | **Identified sequence in tiRNA&tRF library** | **Remaining sequence from parental tRNA sequence** | **potential cleavage region^1^** | **tRNA cleavage region location** |
| --- | --- | --- | --- | --- | --- | --- | --- |
| WT vs KO | 3'tiR_088_LysCTT (n) | LysCTT | 3-half | CUUAAUCUCAGGGUCGUGGGUUCGAGCCCCACGUUGGGCGCCA | GCCCGGCUAGCUCAGUCGGUAGAGCAUGGGACU | GGA(CUCUUAA) | Anticodon loop |
|  | tiRNA-5033-LysTTT-1 | LysTTT | 5-half | GCCCGGAUAGCUCAGUCGGUAGAGCAUCAGACU | UUUAAUCUGAGGGUCCAGGGUUCAAGUCCCUGUUCGGGCG | AGA(CUUUUAA) | Anticodon loop |
|  | 1039 | Pre-ArgCCT | tRF-1 | UCGAGAGGGGCUGUGCUCGCAAGGUUUCUUU | CCAAAGCA | AAGCAUCGAG | Acceptor stem |
|  | 3002A | ProCGG, AGG, TGG, CGG | tRF-3 | AUCCCGGACGAGCCCCCA | GGCUCGUUGGUCUAGGGGUGUGGUUCUCGCUUAGGGCGGGAGACCCAAGAGGUCCCGGGUUCAA | (UUCAAAU)CCC | T-Loop |
|  | 5028/29A | GluTCC | tRF-5 | UCCCACAUGGUCUAGCGG | UUAGGAUUCCUGGUUUUCACCCAGGUGGCCCGGGUUCGACUCCCGGUAUGGGAA | (AGCGGUUA)GG | D-loop |
| KO+RSV vs WT+RSV | 3'tiR_060_MetCAT (n) | MetCAT | 3-half | UCAUAAUCUGAAGGUCGUGAGUUCGAUCCUCACACGGGGCACCA | GCCCUCUUAGUGCAGCUGGCAGCGCGUCAGUU | CAGU(UUCAUAA) | Anticodon loop |
|  | TRF62 | MetCAT | itRF | AGCAGAGUGGCGCAGCGGAAGCGUGCUGG | GCCCAUAACCCAGAGGUCGAUGGAUCGAAACCAUCCUCUGCUA | GCUGGG(CCCAUAA) | D loop |
|  | TRF315 | LysCTT | itRF | GCCCGGCUAGCUCAGUCGGUAGAGCAUGG | GACUCUUAAUCUCAGGGUCGUGGGUUCGAGCCCCACGUUGGGCG | CAUGGGA(CUCUUA) | D loop |
|  | TRF353 | GlnTTG | itRF | GGCCCCAUGGUGUAAUGGUUAGCACUCUGGA | CUUUGAAUCCAGCAAUCCGAGUUCGAAUCUCGGUGGGACCU | CUGGA(CUUUGAA) | D loop |
|  | TRF419 | LeuTAG | itRF | GGUAGUGUGGCCGAGCGGUCUAAGGCGCUG | GAUUUAGGCUCCAGUCUCUUCGGAGGCGUGGGUUCGAAUCCCACCGCUGCCA | CGCUGGA(UUUAGG) | D loop |
|  | tiRNA-5030-LysCTT-2 | LysCTT | 5-half | GCCCGGCUAGCUCAGUCGGUAGAGCAUGGG | ACUCUUAAUCUCAGGGUCGUGGGUUCGAGCCCCACGUUGGGCG | AUGGGA(CUCUUAA) | Stem near Anticodon loop |
|  | tiRNA-5031-HisGTG-1 | HisGTG | 5-half | GCCGUGAUCGUAUAGUGGUUAGUACUCUGCG | UUGUGGCCGCAGCAACCUCGGUUCGAAUCCGAGUCACGGCA | CUGCG(UUGUGGC) | Stem near Anticodon loop |
|  | tiRNA-5031-GluCTC-1 | GluCTC | 5-half | UCCCUGGUGGUCUAGUGGUUAGGAUUCGGCG | CUCUCACCGCCGCGGCCCGGGUUCGAUUCCCGGUCAGGAAA  CUCUCACCGCCGCGGCCCGGGUUCGAUUCCCGGUCAGGGAA | CGGCG(CUCUCAC) | Stem near Anticodon loop |
|  | 1001 | Pre-SerTGA | tRF-1 | GAAGCGGGUGCUCUUAUUUU | CUCGCUGCG | CUGCGGAAGC | Acceptor stem |
|  | 1013 | Pre-AlaCGC | tRF-1 | GGCGAUCACGUAGAUUUU | UCUCCA | CUCCAGGCGA | Acceptor stem |
|  | 1039 | Pre-ArgCCT | tRF-1 | UCGAGAGGGGCUGUGCUCGCAAGGUUUCUUU | CTGGGGU | GGGGUUCGAG | Acceptor stem |
|  | 3004B | GlnTTG | tRF-3 | UCAAAUCUCGGUGGGACCUCCA | GGCCCCAUGGUGUAAUGGUUAGCACUCUGGACUUUGAAUCCAGCGAUCCGAGU | CGAGU(UCAAAU) | T loop |
|  | 3006B | LysTTT | tRF-3 | UCAAGUCCCUGUUCGGGCGCCA | GCCCGGAUAGCUCAGUCGGUAGAGCAUCAGACUUUUAAUCUGAGGGUCCGGGGU | GGGG(UUCAAGU) | T loop |
|  | 3016/18/22B | LysCTT/MetCAT/IleAAT | tRF-3 | UCGAGCCCCACGUUGGGCGCCA | GCCCGGCUAGCUCAGUCGGUAGAGCAUGGGACUCUUAAUCUCAGGGUCGUGGGU | UGGG(UUCGAGC) | T loop |
|  | 5024A | LeuTAA | tRF-5 | GUUAAGAUGGCAGA | ACCUGGCAGUUUCAUAAAACUUAAAGUUUAUAAUCAGAGGUUCAACUCCUCUUCUUAACA | GCAG(AACCUGGCA) | D loop |
|  | 5032A | AspGTC | tRF-5 | UCCUCGUUAGUAUAGUGG | UGAGUGUCCCCGUCUGUCACGCGGGAGACCGGGGUUCGAUUCCCCGACGGGGAG | (AGUGGUGA)GU | D loop |
|  | TRF356/359 | ArgCCG/ArgTCG | tRF-5 | GGCCGCGUGGCCUAAUGGA | UAAGGCGUCUGACUUCGGAUCAGAAGAUUGCAGGUUCGAGUCCUGCCGCGGUCG | (AAUGGAUA)AGG | D loop |
|  | TRF366 | Thr-TGT | tRF-5 | GGCUCCAUAGCUCAGUGGUUAGAGCA | CUGGUCUUGUAAACCAGGGGUCGCGAGUUCGAUCCUCGCUGGGGCCU | (AGUGGUUA)GAGCACUGGU | D loop |
|  | TRF365 | Thr-TGT | tRF-5 | GGCUCCAUAGCUCAGGGGU | UAGAGCGCUGGUCUUGUAAACCAGGGGUCGCGAGUUCAAUUCUCGCUGGGGCCU | (AGGGGUUA)GAG | D loop |
|  | TRF396 | AlaAGC | tRF-5 | GGGGGUAUAGCUCAGCGGU | AGAGCGCGUGCUUAGCAUGCACGAGGUCCUGGGUUCAAUCCCCAAUACCUCCA | (AGCGGUA)GAGC | D loop |
|  | TRF457 | SerAGA/SrTGA | tRF-5 | GUAGUCGUGGCCGAGUGG | UUAAGGCGAUGGACUAGAAAUCCAUUGGGGUUUCCCCACGCAGGUUCGAAUCCUGCCGACUACG | (GAGUGGUUAA)G | D loop |
|  | TRF550/551 |  | tRF-5 | UCCUUGGUGGUCUAGUGGCUAG | GAUUCGGCGCUUUCACCGCCGCGGCCCGGGUUCGAUUCCCGGCCAGGGAA | (AGUGGCUA)GGAUC | D loop |
| RSV vs WT | tiRNA-5034-ValCAC-3 | ValCAC | 5-half | GUUUCCGUAGUGUAGCGGUUAUCACAUUCGCCUC | ACACGCGAAAGGUCCCCGGUUUGAAACCAGGCGGAAACA | GC(CUCACAC)G | Anticodon loop |
|  | tiRNA-5029-ProAGG | ProAGG | 5-half | GGCUCGUUGGUCUAGGGGUAUGAUUCUCG | CUUAGGGUGCGAGAGGUCCCGGGUUCAAAUCCCGGACGAGCCC | UCUCGC(UUAGGGU) | Anticodon loop |
|  | TRF354 | ThrTGT | tRF-5 | GGCCCUAUAGCUCAGGGG | UUAGAGCACUGGUCUUGUAAACCAGGGGUCGCGAGUUCAAAUCUCGCUGGGGCCU | (AGGGGUUA)GA | D loop |
|  | TRF374 | ThrCGT | tRF-5 | GGCUCUGUGGCUUAGUUGGC | UAAAGCGCCUGUCUCGUAAACAGGAGAUCCUGGGUUCGAAUCCCAGCGGGGCCU | (AGUUGGCUA)AAG | D loop |

^1^the potential cleavage region of the parental tRNAs are inferred according to the sequences detected in the tiRNA&tRFs library screening. Naming is directly taken from the tiRNA&tRFs library. Complementarily, we include in the next column the remaining tRNA sequence from the parental tRNA. The first 5 bases from tRNA fragments at the 5’ and 3’ sides are coloured in red and blue, respectively. The full region corresponding to specific loops in the parental tRNA is included and enclosed by parenthesis. When more than one sequence is found for the same identifier, the first one is taken (in no case, the punctual base differences are affecting the cleavage region). Information for tRNAs sequences was taken from GtRNAdb, MINTbase, tRNAdb and tRFdb databases.

**S5 Table. Comparison of cleavage specificity for RNA synthetic substrates *in vitro* and ncRNA in cell assays for other RNaseA superfamily members**.

| **RNase** | **Synthetic substrate** | **Base preference** | **Ref.** | **ncRNA** | **Base preference** | **Ref.** |
| --- | --- | --- | --- | --- | --- | --- |
| **RNase5** |  | **B1** |  |  |  |  |
|  | CpA/UpA | C/U ~12 | 1,2 | Selected tRNA (anticodon loops) |  | 3 |
|  |  |  |  | CA and CU (anticodon loops) |  | 4 |
|  |  | **B2** |  |  |  |  |
|  | CpA/CpG | A/G ~3 | 1,2 |  |  |  |
|  | CpG/CpC | G/C ~3 | 1 |  |  |  |
|  |  |  |  | tRNA (Anticodon loops) | A>U | 4 |
| ***Rana pipiens* RNase (Onconase)** |  | **B1** |  |  |  |  |
|  | UpG/CpG | U/C ~10 | 5 | tRNA | Major: UG↓G  (Variable loop and D arm) | 6, 7 |
|  |  |  |  | tRNA | Minor:  G↓U  C↓U | 6,7 |
|  | polyU/polyC | U/C ~100 | 8 |  |  |  |
|  | tetranucleotide | U/C ~60 | 8 |  |  |  |
|  |  | **B2** |  |  |  |  |
|  | Tetranucleotide | G/A ~800 | 8 |  |  |  |

^1^ Shapiro R. Structural features that determine the enzymatic potency and specificity of human angiogenin: threonine-80 and residues 58-70 and 116-123. *Biochemistry*. 1998; 37: 6847-6856. doi: 10.1021/bi9800146.

^2^ Shapiro R, Riordan JF, Vallee BL. Characteristic ribonucleolytic activity of human angiogenin. *Biochemistry*. 1986; 25: 3527-3532. doi: 10.1021/bi00360a008.

^3^ Su Z, Kuscu C, Malik A, Shibata E, Dutta A. Angiogenin generates specific stress-induced tRNA halves and is not involved in tRF-3-mediated gene silencing. *J Biol Chem*. 2019; 294 16930-16941. doi: 10.1074/jbc.RA119.009272.

^4^ Shigematsu M, Kirino Y. Oxidative stress enhances the expression of 2',3'-cyclic phosphate-containing RNAs. *RNA Biol*. 2020; 17 :1060-1069. doi: 10.1080/15476286.2020.1766861.

^5^ Ardelt W, Lee HS, Randolph G, Viera A, Mikulski SM, Shogen K. Enzymatic characterization of onconase, a novel ribonuclease with anti-tumor activity. *Protein Sci*. 1994; 3: 137.

^6^ Suhasini AN, Sirdeshmukh R. Transfer RNA cleavages by onconase reveal unusual cleavage sites*. J Biol Chem*. 2006; 281: 12201-12209. doi: 10.1074/jbc.M504488200.

^7^ Suhasini AN, Sirdeshmukh R. Onconase action on tRNA(Lys3), the primer for HIV-1 reverse transcription. *Biochem Biophys Res Commun*. 2007; 363 :304-309. doi: 10.1016/j.bbrc.2007.08.157.

^8^ Ardelt W, Ardelt B, Darzynkiewicz Z. Ribonucleases as potential modalities in anticancer therapy. *Eur J Pharmacol*. 2009; 625: 181-9. doi: 10.1016/j.ejphar.2009.06.067.

**S6 Table. Primers used for PCR.**

| **Name** | **Sequences** |
| --- | --- |
| Short-Fw | CCACCGGGTCTTCGAAGACTGTTTG |
| Short-Rv | CATGGGTGGCCCAGAACCTTCTGACAAACGATC |
| Cherry-Kosak-AgeI | TATAACCGGTGCCACCATGGTGAGCAAGGGCGAGGA |
| Cherry-BamHI | ATAAGGATCCCTTGTACAGCTCGTCCATGC |
| GFP-BsrGI | ATATTGTACAGAGGGCAGAGGAAGTCTGCT |
| GFP-EcoRI | TTAGAATTCTTACAGCTCGTCCATGCCGAGAG |
| RSV-A-Fw | CTCAATTTCCTCACTTCTCCAGTGT |
| RSV-A-Rv | CTTGATTCCTCGGTGTACCTCTGT |
| RSV-A-Probe | TCCCATTATGCCTAGGCCAGCAGCA |

The fluorogenic probe was labelled with 6-carboxyfluorescein (FAM) and 6-carboxytetramethylrhodamine (TAMRA).

**S7 Table. Primers and sequences used for PCR of IVT.**

|  | **Sequence (single strand 5’-3’)** | **Primer (+)** | **Primer (-)** |
| --- | --- | --- | --- |
| tRNA Lys^TTT^ | AATATAATACGACTCACTATAGGGCGGATAGCTCAGTCGGTAGAGCATCAGACTTTTAATCTGAGGGTCCAGGGTTCAAGTCCCTGTTCGCCCG | AATATAATACGACTCACTATAGGGCGGATAGC | CGGGCGAACAGGGACTTGAA |
| tRNA Lys^CTT^ | AATATAATACGACTCACTATAGGGCGGCTAGCTCAGTCGGTAGAGCATGGGACTCTTAATCTCAGGGTCGTGGGTTCGAGCCCCACGTTGCCCGCCA | AATATAATACGACTCACTATAGGGCGGC | TGGCGGGCAACGTGGG |
| tRNA Met^CAT^ | AATATAATACGACTCACTATAGGGCTCTTAGTGCAGCTGGCAGCGCGTCAGTTTCATAATCTGAAGGTCGTGAGTTCGATCCTCACACGGCCCACC | AATATAATACGACTCACTATAGGGCTCTTAGTGC | GGTGGGCCGTGTGAGGATCG |
| tRNA Ala^AGC^ | AATATAATACGACTCACTATAGGGGGTGTAGCTCAGTGGTAGAGCGCGTGCTTAGCATGCACGAGGCCCCGGGTTCAATCCCCGGCACCTCCACCC | AATATAATACGACTCACTATAGGGGGTGTAG | GGGTGGAGGTGCC |
| tRNA Asp^GTC^ | AATATAATACGACTCACTATAGGGTCGTTAGTATAGTGGTGAGTATCCCCGCCTGTCACGCGGGAGACCGGGGTTCGATTCCCCGACGGCCC | AATATAATACGACTCACTATAGGGTCGTTAGTATAG | GGGCCGTCGGGGAATC |
